# Supplementary material for: Approaches for Inclusion Complexes of Ezetimibe with Cyclodextrins: Strategies for Solubility Enhancement and Interaction Analysis via Molecular Docking
Source: Int J Mol Sci. 2025 Feb 16;26(4):1686. doi: 10.3390/ijms26041686 (PMC11855275; doi:10.3390/ijms26041686)
Supplement: Supplementary file 1 [file ijms-26-01686-s001.zip › ijms-3413122-supplementary.pdf]

Supplementary Information

Approaches for Inclusion Complexes of Ezetimibe with Cyclodextrins: Strategies for Solubility Enhancement and Interaction Analysis via Molecular Docking

Dae Yeong Cho [1], Jeong Gyun Lee [1], Moon Jung Kim [1], Hyuk Jun Cho [2], Jung Hyun Cho [3,*] and Kyeong Soo Kim [1,*]

[1] Department of Pharmaceutical Engineering, Gyeongsang National University, 33 Dongjin-ro,

Jinju 52725, Republic of Korea

[2] Department of Innovative Drug Discovery and Development, College of Pharmacy, Keimyung University, 1095 Dalgubeoldaero, Dalseo-gu, Daegu 42601, Republic of Korea

[3] Department of Pharmaceutical Engineering, Dankook University, 119 Dandae-ro, Dongnam-gu, Cheonan 31116, Republic of Korea

*Corresponding author: Kyeong Soo Kim, Prof. Dr.
Tel: +82-55-772-339
Fax: +82-55-772-3399
E-mail: soyoyu79@gnu.ac.kr

Jung Hyun Cho, Prof. Dr.
Tel: +82-
Fax: +82-
E-mail: jhcho7301@dankook.ac.kr

Table S1. Data for the Job's plot performed by UV-Visible spectroscopy for aqueous EZT/RM-β-CD.

RM- β-CD (mL)	EZT (mL)	RM- β-CD (nM)	EZT (nM)	R [EZT]/([EZT]+[RM-β-CD])	Absorbance (A)	ΔA	ΔA*R [EZT]/([EZT]+[RM-β-CD])
1	0	40	0	0	0	0.920	0
0.9	0.1	36	4	0.1	0.072	0.848	0.085
0.8	0.2	32	8	0.2	0.182	0.738	0.148
0.7	0.3	28	12	0.3	0.266	0.654	0.196
0.6	0.4	24	16	0.4	0.374	0.546	0.218
0.5	0.5	20	20	0.5	0.447	0.473	0.237
0.4	0.6	16	24	0.6	0.549	0.371	0.223
0.3	0.7	12	28	0.7	0.623	0.297	0.208
0.2	0.8	8	32	0.8	0.733	0.187	0.150
0.1	0.9	4	36	0.9	0.825	0.095	0.086
0	1	0	40	1	0.920	0	0

Table S2. Data for the Job's plot performed by UV-Visible spectroscopy for aqueous EZT/DM-β-CD.

DM- β-CD (mL)	EZT (mL)	DM- β-CD (nM)	EZT (nM)	R [EZT]/([EZT]+[DM-β-CD])	Absorbance (A)	ΔA	ΔA*R [EZT]/([EZT]+[DM-β-CD])
1	0	40	0	0	0	0.918	0
0.9	0.1	36	4	0.1	0.084	0.834	0.0834
0.8	0.2	32	8	0.2	0.173	0.745	0.149
0.7	0.3	28	12	0.3	0.265	0.653	0.1959
0.6	0.4	24	16	0.4	0.358	0.56	0.224
0.5	0.5	20	20	0.5	0.455	0.463	0.2315
0.4	0.6	16	24	0.6	0.541	0.377	0.2262
0.3	0.7	12	28	0.7	0.636	0.282	0.1974
0.2	0.8	8	32	0.8	0.738	0.18	0.144
0.1	0.9	4	36	0.9	0.824	0.094	0.0846
0	1	0	40	1	0.918	0	0

Table S3. Data for the Job's plot performed by UV-Visible spectroscopy for aqueous EZT/HP-β-CD.

HP- β-CD (mL)	EZT (mL)	HP- β-CD (nM)	EZT (nM)	R [EZT]/([EZT]+[HP-β-CD])	Absorbance (A)	ΔA	ΔA*R [EZT]/([EZT]+[HP-β-CD])
1	0	40	0	0	0	0.941	0
0.9	0.1	36	4	0.1	0.105	0.836	0.0836
0.8	0.2	32	8	0.2	0.2	0.741	0.1482
0.7	0.3	28	12	0.3	0.294	0.647	0.1941
0.6	0.4	24	16	0.4	0.387	0.554	0.2216
0.5	0.5	20	20	0.5	0.48	0.461	0.2305
0.4	0.6	16	24	0.6	0.572	0.369	0.2214
0.3	0.7	12	28	0.7	0.663	0.278	0.1946
0.2	0.8	8	32	0.8	0.751	0.19	0.152
0.1	0.9	4	36	0.9	0.854	0.087	0.0783
0	1	0	40	1	0.941	0	0

Table S4. Data for the Job's plot performed by UV-Visible spectroscopy for aqueous EZT/SBE-β-CD.

SBE-β-CD (mL)	EZT (mL)	SBE-β-CD (nM)	EZT (nM)	R [EZT]/([EZT]+[SBE-β-CD])	Absorbance (A)	ΔA	ΔA*R [EZT]/([EZT]+[SBE-β-CD])
1	0	40	0	0	0	0.931	0
0.9	0.1	36	4	0.1	0.102	0.829	0.0829
0.8	0.2	32	8	0.2	0.189	0.742	0.1484
0.7	0.3	28	12	0.3	0.282	0.649	0.1947
0.6	0.4	24	16	0.4	0.373	0.558	0.2232
0.5	0.5	20	20	0.5	0.466	0.465	0.2325
0.4	0.6	16	24	0.6	0.545	0.386	0.2316
0.3	0.7	12	28	0.7	0.647	0.284	0.1988
0.2	0.8	8	32	0.8	0.745	0.186	0.1488
0.1	0.9	4	36	0.9	0.84	0.091	0.0819
0	1	0	40	1	0.931	0	0

Table S5. Calculated binding energies of different docked conformations of EZT/RM-β-CD inclusion complex

Rank	Final Intermolecular Energy (kcal/mol)	Vander waal + H-bond + desolv Energy (kcal/mol)	Electrostatic Energy (kcal/mol)	Final Total Internal Energy (kcal/mol)	Torsional Free Energy (kcal/mol)	Unbound System's Energy (kcal/mol)	Estimated Free Energy of Binding (kcal/mol)
1	-10.84	-10.74	-0.10	-1.47	+2.39	-1.47	-8.46
2	-10.40	-10.32	-0.08	-2.67	+2.39	-2.67	-8.01
3	-10.01	-9.96	-0.06	-1.85	+2.39	-1.85	-7.63
4	-9.87	-9.82	-0.05	-1.78	+2.39	-1.78	-7.48
5	-9.86	-9.85	-0.01	-2.67	+2.39	-2.67	-7.47

Table S6. Calculated binding energies of different docked conformations of EZT/DM-β-CD inclusion complex

Rank	Final Intermolecular Energy (kcal/mol)	Vander waal + H-bond + desolv Energy (kcal/mol)	Electrostatic Energy (kcal/mol)	Final Total Internal Energy (kcal/mol)	Torsional Free Energy (kcal/mol)	Unbound System's Energy (kcal/mol)	Estimated Free Energy of Binding (kcal/mol)
1	-10.83	-10.72	-0.12	-2.23	+2.39	-2.23	-8.45
2	-10.74	-10.65	-0.09	-2.40	+2.39	-2.40	-8.35
3	-10.72	-10.65	-0.06	-2.39	+2.39	-2.39	-8.33
4	-10.71	-10.64	-0.07	-2.42	+2.39	-2.42	-8.32
5	-10.68	-10.58	-0.10	-2.44	+2.39	-2.44	-8.29

Table S7. Calculated binding energies of different docked conformations of EZT/HP-β-CD inclusion complex

Rank	Final Intermolecular Energy (kcal/mol)	Vander waal + H-bond + desolv Energy (kcal/mol)	Electrostatic Energy (kcal/mol)	Final Total Internal Energy (kcal/mol)	Torsional Free Energy (kcal/mol)	Unbound System's Energy (kcal/mol)	Estimated Free Energy of Binding (kcal/mol)
1	-10.29	-10.31	+0.02	-1.66	+2.39	-1.66	-7.90
2	-10.09	-10.10	+0.01	-1.61	+2.39	-1.61	-7.70
3	-10.00	-9.88	-0.12	-1.52	+2.39	-1.52	-7.61
4	-9.61	-9.59	-0.02	-2.36	+2.39	-2.36	-7.22
5	-9.37	-9.28	-0.09	-1.63	+2.39	-1.63	-6.99

Table S8. Calculated binding energies of different docked conformations of EZT/SBE-β-CD inclusion complex

Rank	Final Intermolecular Energy (kcal/mol)	Vander waal + H-bond + desolv Energy (kcal/mol)	Electrostatic Energy (kcal/mol)	Final Total Internal Energy (kcal/mol)	Torsional Free Energy (kcal/mol)	Unbound System's Energy (kcal/mol)	Estimated Free Energy of Binding (kcal/mol)
1	-8.80	-8.60	-0.21	-2.40	+2.39	-2.40	-6.42
2	-8.75	-8.67	-0.08	-2.42	+2.39	-2.42	-6.36
3	-8.58	-8.55	-0.03	-2.41	+2.39	-2.41	-6.19
4	-8.45	-8.30	-0.16	-1.67	+2.39	-1.67	-6.07
5	-8.41	-8.32	-0.10	-2.44	+2.39	-2.44	-6.03
